# Supplementary material for: Are carbon emissions trading and green financial instruments synergistic? -Comprehensive quantitative research based on content analysis
Source: PLoS One. 2024 Mar 7;19(3):e0298601. doi: 10.1371/journal.pone.0298601 (PMC10919601; doi:10.1371/journal.pone.0298601)
Supplement: S1 Data — (ZIP) [file pone.0298601.s001.zip › Data/地级市Z_F工作报告.zip!/┴╔─■/┤≤┴1⁄4╩╨/└┤╫╘╣1⁄2╓┌║┼í╛┬φ┐╦╩2╛▌═°í┐_2014.docx]

各位代表：

　　现在，我代表市政府向大会报告工作，请予审议，并请市政协委员和其他列席人员提出意见。

　　一、2013年政府工作回顾

　　过去的一年，是全面贯彻落实党的十八大精神的第一年。在党中央、国务院和省委、省政府及市委的正确领导下，市政府团结带领全市人民，积极应对复杂严峻的外部环境和经济下行压力增大的挑战，以提高经济增长质量和效益为目标，加快结构调整，持续改善民生，较好地完成十五届人大一次会议确定的各项任务，实现了新一届政府良好开局。

　　预计全市实现地区生产总值7820亿元，增长11%；公共财政收入850.01亿元，增长13.3%；固定资产投资6750亿元，增长20%；社会消费品零售总额2513亿元，增长13%；城市居民人均可支配收入30238元、农村居民人均纯收入17717元，分别增长9.8%和10.8%；城镇登记失业率控制在2.61%。经济实现逆势发展，保持了区域竞争的优势地位。

　　（一）经济发展质量效益提升

　　投资拉动效应强劲。全市共实施亿元以上重大项目1028项，总投资1.58万亿元。城市地铁、丹大快速铁路、南部滨海大道、恒力石化二期等一批重大基础设施和产业项目加快推进。完成亿元以上重大建设项目投资3800亿元，拉动全社会投资增长10.5%。

　　优势产业提质增效。“一企一策”帮助10大行业企业解决困难和问题。东风日产、北车旅顺基地等100个大项目加快推进，红沿河核电1号机组、德豪光电一期等大项目竣工投产，奇瑞汽车瑞虎5下线，恒力石化主营业务收入超300亿元。19个省重点产业集群实现销售收入7300亿元，增长18%，其中6个规模达到500亿元以上。全市完成规模以上工业增加值3180亿元，增长11%。

　　战略性新兴产业蓬勃发展。海洋工程、核电装备生产能力居全国首位。发展物联网、智能装备制造等107个创新驱动项目，137项核心技术和重点产品研制取得重大进展。万人有效发明专利拥有量5577件，增长21%。规模以上工业企业实现高新技术产品增加值增长20%以上。

　　现代服务业不断壮大。高新区、生态科技创新城聚集多家世界500强企业研发和设计中心。恒隆广场等10大项目加快建设，市区集聚企业总部115家，“亿元楼”增至8座。旅游总收入910亿元，增长18.6%。服务业增加值3280亿元，增长9.2%。

　　房地产业和建筑业保持平稳。完成房地产开发投资和销售额分别增长22%和15%；建筑业产值突破2300亿元，增长13.3%。

　　非公经济快步发展。实施中小微企业成长和创新型中小企业培育两项工程，省级创新型中小企业达到103户。民营经济占GDP的比重达到70%。

　　现代农业持续发展。都市型现代农业全面推进，新发展设施农业11万亩、海洋牧场10.2万亩，10个都市型农业园区建设加快。中国农科院都市农业研究所落户普湾新区。肉蛋奶和果菜产量持续增长，粮食产量实现“十一连丰”。

　　（二）全域城市化发挥引擎作用

　　新市区组团释放强大能量。地区生产总值、财政收入、固定资产投资增幅均高于全市平均水平，形成引领辽宁沿海经济带发展、带动东北全面振兴的重要增长极。汽车物流城产业链企业达到150家，大孤山石化产业集群突破1000亿元大关。

　　渤黄海沿岸产业发展提速。推动县域经济进一步壮大，瓦房店“轴承之都”、普湾新区“西装名城”建设迈出重要步伐，庄河循环产业、新兴产业和海洋产业3个经济区完成基础设施建设，花园口新材料、生物产业基地新开工项目47个，长海现代海洋牧场、国际旅游避暑度假区建设提档升级。

　　主城区辐射力持续增强。大连金融服务区、西岗北部沿海商务区、西安路商务商贸区、甘井子 “两城四区”、高新区“一带多园”、旅顺口 “五城一都”等高端化、集约化发展。主城区已成为吸引人才汇聚、创新能力外溢、引领城市转型的动力源。

　　中心镇建设有序推进。投入21亿元推进重点区域和4个中心镇基础设施及公共服务体系建设，太平湾、皮杨、大郑、广鹿岛已成为全域城市化的新亮点。

　　（三）城市功能全面提升

　　“三个中心”建设步伐加快。港口货物吞吐量达4亿吨，集装箱吞吐量突破1000万标箱。大连国际综合物流园、香炉礁物流园等10大物流园区加快建设；承担着东北地区64%的进出口货物吞吐量，完成社会物流增加值800亿元，增长16%。

　　在东北地区率先设立股权交易市场，本外币贷款余额突破万亿元，预计金融增加值占GDP比重7%，新设金融机构33家，新增融资2660亿元，金融中心指数列同类城市第6位。大商所上市焦煤、铁矿石等五大新品种，交易量占全国37.6%，获评中国最佳期货交易所，抢占了全球金融领域一个新的制高点。

　　重大基础设施加速布局建设。全域实施125个城建项目，完成投资424亿元，增长54%。体育中心及周边环境建设圆满完成，金普城际铁路等一批工程加快推进，长山大桥合龙，202路快轨延伸线试通车。大伙房水库输水入连、长海跨海引水工程具备通水能力。地铁1、2号线完成工程量85.2%。新机场前期工程加快推进，沿岸商务区累计完成投资45.7亿元。东港商务区建设取得阶段性成果，国际会议中心投入使用。

　　城乡环境不断优化。全年造林绿化90.8万亩，城市绿化覆盖率达到45.3%。完成44个国家及省级生态乡镇、101个生态村创建。新建农村公路424公里、饮水工程40项，289个村实施新农村“六化”工程建设。完成城市建设“五个一”和县城建设“三个一”工程项目111个。万元GDP能耗降低3.8%，主要污染物平均减排3%以上。

　　创新城市管理成效显著。我市成为全国首批“智慧城市”、“水生态文明试点示范城市”，“无线城市”建设、“三网融合”、“智慧旅游”加速推进。创新建立社会组织直接登记和“一业多会”机制。改善社区工作人员待遇，促进基层组织自治和社会管理水平全面提升。提高防灾减灾救灾能力。探索建立“三位一体”志愿服务、“365”、“帮万家”等社会服务新模式。深化中心城区环卫体制改革，城市环境质量提升。建立打击食品药品制假售假违法行为联动机制，确保食品药品安全。我市被评为全国“社会管理综合治理优秀市”。

　　（四）改革开放活力持续增强

　　重点领域改革不断深化。全市取消政府行政许可事项17项，下放市级审批管理权限41项，高新区成为全省首个“无行政收费区”。积极推进“营改增”试点，9.41万户企业受益。推进北三市公立医院改革试点，取消“以药养医”。装备投资集团、金重集团组建完成，交运集团等国有企业完成股权多元化改革。

　　全域开放成果丰硕。长兴岛石化产业园区被确定为国家重点支持的产业基地，旅顺开发区晋位国家级，金普新区、大连自由贸易园（港）区正在积极申办。出台《关于加快推进沿海经济带重点园区发展的若干意见》，推动重点园区成为全域开发开放的主战场。出口市场扩展到全球196个国家和地区，外贸出口367.5亿美元、增长9.1%。实际使用外资136亿美元，连续5年居副省级城市前列。实际到位内资2426亿元，总量继续保持全省第一。19个省重点园区新签约落地3000万美元以上外资项目40个，内资亿元以上项目280个。世界500强企业入连达109家，投资项目238个。民营企业成为出口的主力军，增幅达到40.8%。成功承办2013夏季达沃斯年会，城市影响力和美誉度全面提升。

　　（五）人民群众共享发展成果

　　民生支出大幅增长。就业、教育、社会保障等方面的投入达到712.4亿元，增长26.3%。全面完成15项重点民生工程。

　　积极稳定和扩大就业。实现城镇新增就业23.3万人，创业就业3.3万人，稳定就业率达到67.4%，本地生源高校毕业生就业率99.6%。

　　社会保障水平再上台阶。在全省率先实现“新农保”与“城居保”统一接轨。建立职工医保门诊统筹制度和城镇居民大病医保制度。连续第9年提高企业退休人员养老金水平，城乡居民基础养老金和失业保险待遇标准再次提高。最低工资标准分别达到1300元和1200元。城乡低保分别提高10%和20%，保障标准全国领先。修订医疗救助办法，发放救助资金8104万元。

　　市民居住条件不断改善。全市新建保障房23702套，基本建成21044套。发放个人住房公积金贷款140亿元，4.2万户家庭改善住房条件。加快推进城子坦淹没区、华铜矿塌陷区整体搬迁。实施“暖房子”工程300万平方米，整治弃管楼院31万平方米，近4000户家庭受益。

　　教育事业长足进步。全年净增1.5亿元提高普通高中公用经费标准，5个区通过全国义务教育发展基本均衡区认定。中职教育实现学费全免，农村地区校车配备任务超额完成。新开办公办幼儿园24所，增加学位4700个。

　　医疗软实力有效提升。市急救中心、医大三院、大连大学附属中山医院等项目加快推进，医大一院心血管诊疗中心投入使用，全市整体医疗救治能力增强。新农合最低筹资标准提高，住院支付比例达到75%左右。落实计划生育独生子女伤残死亡家庭扶助政策，近1.2万人受益。

　　文化体育全面发展。普湾新区“三馆”建设加快，大连京剧院新建工程等有序推进。公益性文化场馆全部免费开放，艺术院团演出1400多场、送戏下乡300场。我市成为首批国家“公共文化服务体系示范区”城市、国家级文化和科技融合示范基地。大连文化产业创新园区全面启动，世茂嘉年华等项目快速推进。成功承办“十二运”14项赛事，大连健儿在全运会上获金牌总数全省第一，15座新建场馆为大连文体事业发展奠定了良好基础。

　　社会保持和谐稳定。积极推动积案化解，实现案结事了。加大安全监管力度，安全生产形势保持稳定。应急管理体系不断完善。平安大连、法治大连建设迈出新的步伐。我市获得全国平安建设“长安杯”最高奖。

　　民族宗教、残疾人、外事、侨务、对台、人防、档案、仲裁、双拥共建、民兵预备役、边海防等工作都取得新进步。

　　（六）政府自身建设不断加强

　　作风改进成效明显。深入开展党的群众路线教育实践活动及“作风建设年”活动，认真查摆在“四风”上存在的突出问题，按照为民、务实、清廉的要求，对自查和征求到的意见建议逐项分解，确定20个整改方向、68个整改项目，狠抓整改落实和建章立制，巩固教育实践活动成果。

　　反腐倡廉建设加强。强化廉政监察、执法监察和效能监察，软环境进一步改善。加强对征地拆迁、重大投资项目等领域的监督和领导干部经济责任审计。加大纠风治乱和问责力度，着力解决一批损害人民群众利益、阻碍经济社会发展的突出问题。

　　依法行政能力提升。全面推进依法行政，法治政府建设进入新阶段。公共资源交易中心挂牌运行，民意网和12345市民热线作用突出。积极落实市人大常委会监督意见建议，主动向人大报告工作，重大事项与政协民主协商，认真办理人大代表建议、政协委员提案，自觉接受人大法律和工作监督、政协民主监督、社会各界监督。

　　各位代表，2013年大连经济社会发展的成绩来之不易，凝结着社会各界的心血、智慧和汗水。在此，我代表市政府，向所有关心支持大连经济社会发展的同志们、海内外各界朋友们，致以崇高的敬意和衷心的感谢！

　　实践使我们深切地体会到：大连能够在逆境中砥砺前行、在困难中创新突破，得益于我们坚定不移地贯彻落实党中央、国务院，省委、省政府和市委的科学决策，始终坚持科学发展不动摇，直面挑战，勇于担当，创造了逆势上扬、率先发展的新业绩和新优势；得益于我们立足大连实际，坚持转方式调结构，实施全域城市化发展战略，构建起多点支撑、全域开发开放的新格局，为大连经济社会长期健康发展奠定了坚实基础；得益于我们牢记执政为民的理念，充分调动全市人民支持发展、参与发展的积极性，凝心聚力，团结拼搏，形成了政通人和的大好局面。这些宝贵的精神财富，我们要倍加珍惜，不断发扬光大。

　　回顾一年的工作，我们必须清醒地看到前进道路上的困难和问题：外需、资源和环境对经济发展约束加剧，我市经济企稳向好的基础尚不稳固；制约经济社会发展的体制机制矛盾依然突出，发展还面临不少障碍和困难；工业经济特别是传统优势产业面临较大的转型压力，现代服务业发展亟待加速；城市环境、公共服务能力和社会治理水平与人民群众的期待仍有差距；政府职能转变和自身建设还需进一步加强。对此，我们将高度重视并在今后切实加以解决。

　　二、2014年政府工作目标与任务

　　2014年是贯彻落实党的十八届三中全会精神、全面深化改革的第一年，是完成“十二五”规划目标的关键之年。政府工作的总体思路是：全面贯彻中央总体工作部署和习近平总书记一系列重要讲话精神，落实省委、省政府和市委各项决策要求，坚持稳中求进工作总基调，把改革创新贯穿于经济社会发展各个领域各个环节，坚持“四化”统筹、协调推进，着力转方式调结构，持续改善民生，提升城市功能，切实提高经济发展质量和效益，加快建设产业结构优化的先导区和经济社会发展的先行区。

　　经济社会发展主要预期目标是：地区生产总值增长10%，万元GDP综合能耗下降3.77%，公共财政收入增长10%，固定资产投资增长20%，社会消费品零售总额增长13%，全体居民可支配收入增长11%，城镇登记失业率控制在3%以下。

　　2014年，重点抓好以下六项工作：

　　（一）着力全面深化改革，进一步增强发展活力

　　按照十八届三中全会的总体部署和习近平总书记视察大连时提出的“加快建设产业结构优化的先导区和经济社会发展的先行区”的重要指示精神，落实市委十一届六次全会提出的要求，通过全面深化改革进一步激发内在活力，促进经济持续健康发展、社会和谐稳定。

　　深化经济领域改革。加快以混合所有制为重点的现代产权制度改革，清理、废除各种不合理规定和隐性壁垒，推动非公企业进入公用事业、基础设施等领域。积极推进注册资本认缴登记等工商登记制度改革，建立现代市场体系，加强社会信用体系建设。支持各种非公资本参与国企股权多元化改革，推动瓦轴、金重集团整体上市，组建大连港投融资控股集团。深化地方金融改革，大力发展普惠金融。推进农村改革，进一步明确农村土地所有权、承包权、经营权之间的关系，开展农村土地承包经营权确权登记办证试点，积极稳妥推进土地流转，加快构建新型农业经营体系。实现市场开放到位、政策使用到位、要素配置到位。

　　推进行政管理体制改革。优化机构设置，正确履行职能，完成食品药品管理体制和卫生计生体制改革。创新财政支持产业发展方式，确立企业投资主体地位，增强财政资金的引导和放大作用。围绕“减少、取消、管理、提效、下放”，深化行政审批制度改革。

　　加快社会领域改革。推动事业单位改革，建立事业单位法人治理结构。强化社区自治和服务功能，构建基础综合性服务平台。加大政府购买服务力度，激发社会组织和市场活力。合理配置公共资源，推进公共服务均等化。

　　（二）深入推进全域城市化，争创开发开放新优势

　　打造对外开放新高地。围绕中央城镇化会议精神和国家赋予的战略定位，完善金普新区总体规划，创新体制机制，努力构建面向东北亚区域开放合作的战略平台。积极申办建设大连自由贸易园（港）区，力争长兴岛综合保税区和中日韩循环经济示范基地获得国家批准，抢占对外开放的制高点。

　　提升重点园区功能。加快长兴岛世界级炼化一体化产业基地、瓦房店轴承产业园区、松木岛精细化工园区、庄河大连国家生态工业示范园区、花园口新材料和生物产业基地建设。提升高新区软件和信息技术服务、大连湾装备制造、金州新区装备和电子信息、旅顺交通运输装备、保税区汽车及零部件等产业聚集度。推进太平湾沿海经济区、北黄海新区、长山群岛旅游避暑度假区加快发展。提升主城区东港商务区、梭鱼湾商务区等建设水平。

　　加快城乡发展一体化。走以人为核心的新型城镇化道路，坚持城镇发展与产业成长“两手抓”，着力突破农业转移人口市民化、土地利用高效化和资金投入多元化3个关键点，注重绿色发展和文化传承，统筹建设质量和发展速度。持续推进农村“三新”工程建设，在150个村实施新村庄“六化”工程，新建通屯油路800公里，使广大农民共建共享现代化成果。

　　推动全域开放向纵深发展。加快外贸出口基地建设，努力培育外贸出口新优势。大力引进世界500强和行业百强企业，引导国内外企业在连设立研发中心和总部基地，实际使用外资增长10%，实际到位内资增长15%。加强区域间交流与合作，支持优势企业实施海外投资并购。

　　（三）大力实施创新驱动战略，加快转方式调结构步伐

　　加强科技创新体系建设。加大财政科研经费投入，全社会研发投入占GDP比重不低于2.5%。实施科技创新驱动重大专项，构建重大技术装备、新能源等10个产业协同创新联盟，支持重大技术攻关，力争成为东北地区“创新中心”。加强知识产权保护，提高发明专利转化率。

　　推进产业转型升级。深入实施工业“五项工程”，提升石油化工、船舶制造、汽车及零部件、软件和电子信息等超千亿级产业集群的竞争优势。安排战略性产业项目资金，重点支持战略性新兴产业发展。加大对服务业重点领域、关键环节和新兴业态的政策支持，促进制造业与服务业加快融合，大力发展总部和楼宇经济。争创“全国质量强市示范城市”和“全国知名品牌创建示范区”。

　　强化投资和消费拉动。全市计划安排投资规模1000万元以上建设项目1998个，总投资2.05万亿元。积极培育和引导消费需求，着力推动信息消费、电子商务、商贸旅游等服务业升级，打造邮轮、温泉、海岛、乡村旅游新优势。推动劳动报酬增长和劳动生产率提高同步，持续拓展社会消费能力和空间。增加普通商品住房有效供给，促进房地产业健康平稳发展。

　　加快农业现代化进程。继续稳定粮食生产，抓好“菜篮子”工程，提高优势特色产业发展和农产品质量安全水平。全年新发展设施农业10万亩，建设都市型现代农业园区10个、海洋牧场10万亩。推进农业信息化建设，大力发展生态友好农业，实现农业可持续发展。加强海洋渔业持续健康发展政策支持，建设现代渔业强市。

　　（四）不断强化城市核心功能，进一步优化生态环境

　　加快“三个中心”建设。推进以大窑湾北岸为核心的航运中心建设，优化“一岛三湾”等三大核心港区港口功能，提升大连港的竞争力和辐射力。积极推进大连新机场及沿岸商务区建设，持续增强港航口岸软实力。推进以太平湾为核心的物流中心建设，培育本地重点物流企业10家，引进全球物流运营商2家。推进以大商所为龙头的金融中心建设，组建交易(所)集团。积极发展科技金融、航运金融、绿色金融。实施中小微企业融资促进行动和农村金融倍增计划，完善中小企业和“三农”投融资服务体系。扩大直接融资，全年“新三板”和四板挂牌企业30家。

　　加强城市规划建设。完善城市总体规划和各类专项规划，确保规划的科学性、严肃性。加快南部滨海大道、丹大快速铁路、金普城际铁路、长兴岛铁路二期、渤海大道一期、疏港路拓宽改造、大连至旅顺中部通道、大连湾海底隧道等一批重大交通基础设施建设。加速解决陆岛交通问题。地铁1、2号线试运营，实现大连迈入“地铁时代”。

　　提升城市管理水平。全面实施网格化城市管理，提高城市管理精细化水平和快速反应能力。加快“宽带大连”、“无线城市”建设，提升智慧城市基础功能。

　　加大生态建设与保护力度。继续实施青山生态系统工程，重点治理交通干道两侧及大中型水库周边废弃矿山，全年栽植树木1.2亿株、造林补植60万亩，加强造林绿化养护管理。推进国家水生态文明试点建设。加强海域海岛海岸带生态整治修复，建设国家级海洋公园和海洋特别保护区。实施“蓝天工程”，开展万家企业节能低碳行动，单位工业增加值综合能耗同比降低3%。继续开展国家新能源汽车推广应用工作。完成热电集团、大化集团等燃煤电厂脱硫脱硝设施建设，主要污染物平均减排2%。让大连的山更绿、水更清、天更蓝。

　　（五）切实加强社会和文化建设，持续增进人民福祉

　　扎实做好重点民生工程。把保障和改善民生作为政府工作的出发点和落脚点，高标准实施15项重点民生工程，努力使改革发展成果更多更公平地惠及人民群众。

　　加强就业和社会保障工作。加大高校毕业生、失地失海人员和困难人群的就业工作力度，全年实现城镇新增就业21万人。完善统筹城乡的社会保障体系，将职工基本养老保险与城市居民社会养老保险衔接政策落实到位，做好企业退休人员养老金调整工作。建立城乡居民大病保险制度，积极推进工伤预防试点。进一步提高基本民生保障水平和社会救助能力，增强社会救助兜底功能，实施农村困难家庭危房改造和救助。推动养老、健康服务业规模化、产业化发展。

　　改善城乡居民生活条件。建立新的食品药品安全监管体制和运行机制，保障食品药品安全。完善政府储备和商业储备体系，保持生活必需品市场的平稳运行和物价稳定。推进租赁住房配比建设和租赁市场培育，完成棚户区（危旧房）改造1.98万户，新增租赁住房补贴家庭1.1万户。实施住房维修“一二三”工程，加快物业服务市场化、专业化进程。强化交通环境综合治理，加快停车场建设，更新公交车辆1111台，不断增加交通资源的有效供给。

　　加快发展社会事业。支持在连高校发展，促进高校与地方发展、企业升级充分融合。推动高水平职业院校建设和职业教育集团化办学。深入推进普通高中优质特色发展。促进义务教育全域均衡发展，继续实施标准化学校建设工程，推进农村中小学供暖设施改造，逐步建立和完善中小学生午餐集体用餐服务体系。新开办公办幼儿园15所，增加普惠性学位3000个。继续实施医疗软实力提升工程，推进体育新城卫生中心、市友谊医院急诊楼和大连大学附属新华医院项目建设，实施乡村卫生服务一体化管理。新农合最低筹资标准提高到520元，年人均基本公共卫生服务经费补助标准提高到40元。依法推动“单独”夫妇生育二孩政策落实，推动健康城市建设。

　　提升城市文化品质。制定大连文化发展规划，支持文化创意、数字出版、动漫游戏等产业发展，加强文物和非遗项目保护，推进基层图书馆建设，丰富群众文化生活。加强新建体育场馆管理，广泛开展全民健身运动，促进竞技体育发展。

　　深化社会治理创新。健全社会普法教育机制，完善信访信息化和联合接访制度。推动社会组织管理体制机制改革创新，充分发挥基层组织和社会组织在社会治理中的作用。全面启动全国安全发展示范城市创建工作。完善立体化社会治安防控体系，依法防范和惩治各类违法犯罪活动。加强应急管理体系和联动机制建设，提高处置公共安全危机和突发事件的能力。做好蝉联第四届全国文明城市、创建第八轮全国双拥模范城和第九届社区居委会换届选举工作。

　　继续做好民族宗教、残疾人、外事、侨务、对台、人防、档案、仲裁、防震减灾、民兵预备役、边海防等工作。

　　（六）建设法治政府和服务型政府，树立为民务实清廉良好形象

　　推进法治政府建设。健全科学、民主、依法决策机制和程序，完善决策问责和纠错制度。加强规范性文件、重大决策合法性审查，健全行政执法程序和监督机制。推进行政执法体制改革，重点加强食品药品、安全生产、环境保护等领域基层执法力量。

　　强化政府服务功能。推进市本级行政许可事项“授权到位”，优化流程，提高服务质量。加快政务公开、政府信息公开和行政机关质量管理体系建设，实现电子政务外网全域覆盖。构建公共资源交易监管平台，推动公共资源交易“管办分离”、“阳光交易”。

　　提高勤政廉政水平。严格落实中央和省、市委关于改进工作作风、密切联系群众的各项规定，健全领导干部带头改进作风、深入基层调查研究机制，持之以恒地抓好软环境建设。坚持厉行节约、反对浪费，严格控制“三公”经费支出和楼堂馆所建设，推进公车制度改革。加强对重大投资项目、重大资金支出的监督和跟踪审计，严厉查处违纪违法案件。加强对政府依法行政的监察检查，提高行政效率，保证政令畅通。

　　各位代表，政府的权力来自人民，对人民负责，受人民监督。市政府将自觉接受人大及其常委会的法律监督、工作监督和市政协的民主监督，严格执行市人大决议决定，切实做好建议提案办理工作。广泛听取民主党派、工商联、无党派人士、各人民团体的意见，高度重视社会监督和新闻舆论监督，不断创造让人民群众满意的新业绩。

　　各位代表，新的使命催人奋进。让我们紧密团结在以习近平同志为核心的党中央周围，在市委的坚强领导下，以更加饱满的热情、更加昂扬的斗志、更加扎实的作风，开拓创新，埋头苦干，为谱写好“中国梦”的大连篇章作出新的贡献！
